# Impact of *Azospirillum* sp. B510 Inoculation on Rice-Associated Bacterial Communities in a Paddy Field

**DOI:** 10.1264/jsme2.ME13049

**Published:** 2013-11-19

**Authors:** Zhihua Bao, Kazuhiro Sasaki, Takashi Okubo, Seishi Ikeda, Mizue Anda, Eiko Hanzawa, Kaori Kakizaki, Tadashi Sato, Hisayuki Mitsui, Kiwamu Minamisawa

**Affiliations:** 1 Graduate School of Life Sciences, Tohoku University, Katahira, Aoba-ku, Sendai, Miyagi 980–8577, Japan; 2 National Agricultural Research Center for Hokkaido Region, Shinsei, Memuro-cho, Kasaigun, Hokkaido 082–0081, Japan

**Keywords:** *Azospirillum* sp. B510, inoculation, rice paddy field, pyrosequencing, bacterial community

## Abstract

Rice seedlings were inoculated with *Azospirillum* sp. B510 and transplanted into a paddy field. Growth in terms of tiller numbers and shoot length was significantly increased by inoculation. Principal-coordinates analysis of rice bacterial communities using the 16S rRNA gene showed no overall change from B510 inoculation. However, the abundance of *Veillonellaceae* and *Aurantimonas* significantly increased in the base and shoots, respectively, of B510-inoculated plants. The abundance of *Azospirillum* did not differ between B510-inoculated and uninoculated plants (0.02–0.50%). These results indicate that the application of *Azospirillum* sp. B510 not only enhanced rice growth, but also affected minor rice-associated bacteria.

The genus *Azospirillum*, which includes nitrogen-fixing Gram-negative *Alphaproteobacteria*, has been known for many years to be composed of plant-growth-promoting bacteria. Among the *Alphaproteobacteria*, *Azospirillum* is one of the most studied genera (8). This genus was isolated not only from the rice phyllosphere (7, 17), but also from the rhizosphere of other plants (21). Rice growth was enhanced by inoculation with *Azospirillum* (15).

*Azospirillum* sp. strain B510 was isolated from surface-sterilized stems of field-grown rice (7). Rice tiller numbers and seed yield were significantly increased with B510 inoculation in paddy fields (13). Moreover, Yasuda *et al.* (26) showed that rice plants inoculated with B510 had greater resistance against two diseases caused by the virulent rice blast fungus *Magnaporthe oryzae* and by the virulent bacterial pathogen *Xanthomonas oryzae*. Thus, *Azospirillum* sp. B510 is a promising bacterial inoculant for plant growth promotion and agricultural practices (13).

The plant-growth-promoting effects of *Azospirillum brasilense* Sp245 were due to the production of indole-3-acetic acid (24). *Azospirillum* cells inoculated into rice plants often spread from below-ground roots into above-ground tissues (4). However, *Azosprillium* sp. B510 inoculation gave rise to colonization in basal parts of rice plants in a laboratory experiment (26). Little is known about the effect of *Azospirillum* sp. B510 inoculation on rice-associated bacterial communities in field conditions. The objective of the present study was to determine whether *Azospirillum* sp. B510 inoculation changes rice-associated bacterial communities, including the B510 inoculant, when the effects of inoculation first appear in a paddy field.

Although our previous study clearly indicated that *Azospirillum* sp. B510 tagged with the DsRed gene able to colonize endophytically in rice seedlings (12), we determined the initial colonization level of B510 carrying the plasmid pHC60 (3) in 7-day-old seedlings of *Oryza sativa* cv. Nipponbare (see supplementary material). For the field experiment, *Azospirillum* sp. B510 was cultured in nutrient broth (Difco, Detroit, MI, USA) at 30°C for 16 h. A bacterial suspension (1×10^8^ CFU mL^−1^) was prepared with distilled water. Three-week-old rice seedlings (*Oryza sativa* cv. Nipponbare) were inoculated with 500 mL of the bacterial suspension (final density: 1.5×10^6^ CFU mL^−1^) on a tray containing 300 seedlings (Fig. S1). The seedlings were incubated in a greenhouse for 5 d in 2011 and then transplanted to Kashimadai experimental field (Tohoku University, Japan; 37°28′N, 141°06′E), which had been treated with nitrogen fertilizer (30 kg ha^−1^) (Fig. S1). Overall, 300 seedlings of each cultivar were planted in 3×4.5 m plot. Hills were spaced 30 cm apart. The soil chemical properties were as follows: pH, 5.2; total C content, 4.3%; total N content, 0.1%; Truog phosphorous, 74 mg P_2_O_5_ kg^−1^.

Plant growth (tiller number and shoot length) was measured 46 d after transplanting (DAT) (Fig. S1) (23). Rice plants were sampled at 51 DAT (Fig. S1). Shoot (above-ground plant tissues starting 10 cm above the root) and base (including 10 cm stem and 1 cm root) were separated (Fig. S2), because B510 cells colonized rice basal parts in laboratory experiments (26).

Three sets of composite samples including at least three rice plants were independently prepared for the bases or shoots (approximately 100 g each) in each treatment, and then stored at −80°C until used. The composite samples of bases or shoots were homogenized without surface sterilization to prepare shoot- and base-associated bacterial cells containing both endophytes and epiphytes. DNA was extracted by the bacterial enrichment method (11). Bacterial communities were analyzed by triplicate 454 pyrosequence analyses targeting the V2–V3 region of the 16S rRNA gene using 454 barcode tags (20). Raw pyrosequencing reads were processed using the Quantitative Insights Into Microbial Ecology (QIIME) software package (2). The reads were assigned to each sample according to sample-specific barcodes. Low-quality reads with a length shorter than 300 bp, with an average quality score lower than 25, with mismatching primer sequences, or with ambiguous bases (denoted by N), were eliminated from downstream analyses. The forward and reverse primer sequences were removed from the quality-filtered reads. The remaining sequences were clustered into operational taxonomic units (OTUs) at 97% similarity. Taxonomic assignment of representative OTUs was performed using the RDP Classifier (25) with default parameters. Principal-coordinates analysis (PCoA) was performed using weighted UniFrac distances (14). The project number for 454 pyrosequence reads in the National Center for Biotechnology Information (NCBI) database is ID DRA000965.

*Azospirillum* sp. B510 inoculation significantly enhanced tiller numbers (by 8.6%) and shoot length (3.1%) as compared with uninoculated controls (*t*-test; *P*<0.05; *n*=25) (Fig. S3). This tiller number increase by B510 inoculation is similar to the results of a previous field experiment in Hokkaido, Japan (13).

For bacterial communities, the total number of pyrosequence reads of 16S rRNA genes from base and shoot, with and without B510 inoculation, ranged from 16,156 to 33,906 (Table S1). Bacterial community structures were analyzed by PCoA (Fig. 1). PC1 (79.3%) showed that bacterial communities were separately clustered in the shoot and base. PC2 accounted for only 6.9% of the differences in microbial communities. There were no marked shifts in bacterial communities by B510 inoculation in either the shoot or base of the rice plants (Fig. 1).

The compositions of entire bacterial communities are shown at the class level (Table S2) and with more detailed taxa (Order, Family and Genus) (Table 1). At the class level, inoculation significantly increased the relative (percent) abundance of the class *Negativicutes* (phylum *Firmicutes*) in the rice-plant base (*P*<0.05; *t*-test), although *Alphaproteobacteria* (>30% of total sequences) were the most abundant in both shoot and base communities (Table 1). In the *Alphaproteobacteria*, *Rhizobium* (relative abundance, 9.5–20.7%) and *Methylobacterium* (15.2–22.0%) were dominant bacterial groups in the rice tissue. However, their relative abundances were not significantly influenced by inoculation. In contrast, relatively minor bacterial groups were significantly influenced by B510 inoculation: the relative abundance of *Aurantimonas* increased significantly in the shoot (from 4.8% in controls to 6.3% in inoculated plants) and *Methylocystis* decreased in the base (from 5.0% to 2.9%) with B510 inoculation. Our result was partially consistent with the previous observation that application of *Azosprillium brasilense* strains as inoculants did not influence the dominant members of the endophytic microbial communities in the rice phyllosphere using PCR-DGGE (22). DGGE profiles of the bacterial community might limit our understanding because its resolution is relatively low. However, in this study, minor bacterial groups, such as *Auriontimonas* (4.8% of relative abundance), *Methylocystis* (5%) and members of *Veillonellaceae* (>1%), were significantly influenced by inoculation.

*Aurantimonas* has been found in above-ground parts of plants such as rice, soybean, *Arabidopsis thaliana*, and *Jatropha curcas* (1, 5, 16, 18). Their population was likely controlled in a manner similar to that of legume-rhizobia symbiosis (10). These findings suggest that *Aurantimonas* spp. are ubiquitous in the phyllosphere and have close interactions with plants. *Veillonellaceae* is a single family of anaerobic Gram-negative bacteria within *Firmicutes*, unlike most other members of the *Firmicutes* (19). Members of *Veillonellaceae* have been detected from the human gut and from rice paddy soil (6, 9). Hooda *et al.* (9) observed that consumption of soluble corn fiber led to increases in *Veillonellaceae* in the gut of healthy men. Thus, it is likely that the members of *Aurantimonas* and *Veillonellaceae* are associated with higher organisms, although their biology is largely unknown. Thus, the limited knowledge of bacterial functions makes it difficult to discuss the relationships between the bacterial community and rice growth promotion, although it is possible that growth enhancement was induced by minor bacterial changes.

In the present study, the B510 inoculant and natural *Azospirillum* populations were not differentiated because of the limitation of the pyrosequence read length (approximately 400–500 bp). However, it is possible to estimate the fate of the inoculant during rice growth in the field by observing the fluctuation of *Azospirillum* abundance. The relative abundance of *Azospirillum* was not significantly influenced by B510 inoculation, although it was low (0.02–0.5%) in both the base and shoot (Fig. 2), suggesting that the abundance of the B510 inoculant decreased at least to that of natural *Azospirillum* populations at 51 DAT (Fig. S1). Apart from the inoculation effect, the relative abundance of *Azospirillum* was higher in the base (0.26–0.29%) than in the shoot (0.02–0.04%) (Fig. 2). The initial bacterial community was not determined at 0 DAT in this field experiment. However, 5.9×10^4^ CFU per plant of *Azospirillum* sp. B510 (0.6% of total inoculant of 10^6^) was detected from surface-sterilized plant tissue in the present study, suggesting that *Azospirillum* sp. B510 cells colonized in rice tissue as endophytes just after 0 DAT.

In conclusion, the present study revealed that B510 inoculation significantly influenced minor bacterial groups of the shoot and base of rice plants, but not major bacterial groups. These results indicate that *Azospillium* sp. B510 inoculation not only stimulated rice growth, but also affected relatively minor rice-associated bacteria in the rice base and shoot.

## Supplementary Information



## Figures and Tables

**Fig. 1:**
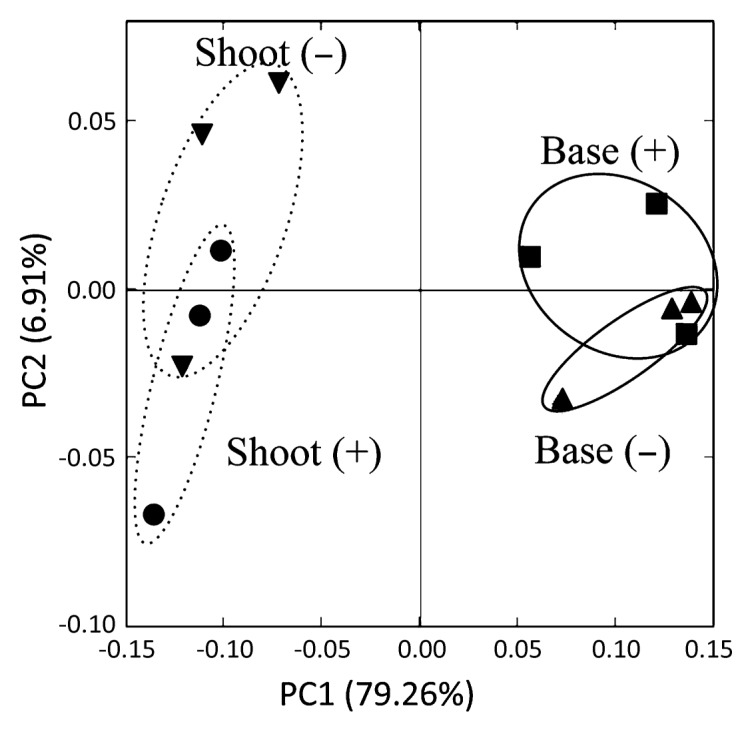
Principal-coordinates analysis (PCoA) of bacterial communities in the base and the shoot of rice plants with (+) and without (−) *Azospirillum* sp. B510 inoculation. ▼, Shoot without inoculation (control); ●, shoot with inoculation; ■, base with inoculation; ▲, base without inoculation (control). Broken line (---): microbial communities of shoot with and without *Azospirillum* sp. B510 inoculation; solid line (—): microbial communities of base with and without *Azospirillum* sp. B510 inoculation.

**Fig. 2:**
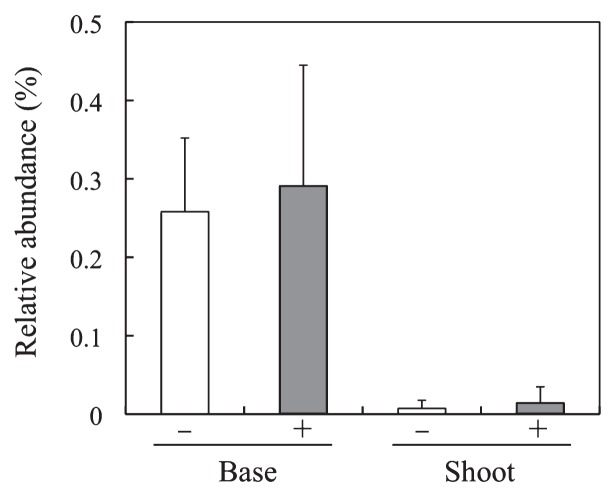
Relative abundance of *Azospirillum* spp. in the rice plant’s base and shoot with (+) and without (−) *Azospirillum* sp. B510 inoculation.

**Table 1:** Relative abundance of the predominant bacterial taxa (as determined from 16S rRNA) in base and shoot of rice plants inoculated with *Azospirillum* sp. B510 (*n*=3 for each sample). Standard deviation is shown in parentheses.

Taxa	Relative abundance (%)			

	Base		Shoot	
	
	−	+	−	+
*Proteobacteria*	53.9 (3.7)	56.0 (9.2)	91.3 (1.6)	91 (2.0)
*Alphaproteobacteria*	38.4 (1.9)	36.8 (6.2)	55.7 (7.6)	55.2 (3.1)
*Rhizobiales*	33.5 (1.5)	31.5 (4.3)	47.5 (5.0)	48.2 (3.1)
*Rhizobium*	9.5 (1.4)	12.6 (0.8)	20.7 (8.3)	16.3 (3.3)
*Methylobacterium*	0.7 (0.2)	0.3 (0.1)	15.2 (1.5)	22.0 (4.1)
*Aurantimonas*	0.2 (0.1)	0.1 (0.03)	4.8 (0.6)	6.3* (1.0)
*Methylocystis*	5.0 (0.4)	2.9* (0.5)	0.4 (0.1)	0.5 (1.0)
*Sphingomonadales*	0.6 (0.1)	0.8 (0.3)	4.3 (2.4)	3.5 (0.7)
*Betaproteobacteria*	7.4 (0.7)	10.7 (2.2)	21.0 (10.4)	11.0 (4.8)
*Burkholderiales*	6.9 (0.7)	9.7 (2.1)	21.0 (10.4)	11.0 (4.8)
*Gammaproteobacteria*	4.3 (3.9)	4.6 (1.6)	13.5 (6.2)	22.9 (10.1)
*Deltaproteobacteria*	1.1 (0.3)	0.9 (0.2)	0.3 (0.2)	1.1 (1.2)
Others	2.7 (0.4)	3.1 (0.7)	0.8 (0.2)	0.8 (0.3)
*Firmicutes*	10.6 (2.1)	15.6 (4.5)	2.9 (0.8)	3.6 (1.5)
*Negativicutes*	1.8 (0.4)	3.3* (0.7)	<0.03	<0.02
*Selenomonadales*	1.8 (0.2)	3.3* (0.3)	<0.03	<0.02
*Veillonellaceae*	1.4 (0.3)	2.7* (0.5)	<0.03	<0.02
*Bacilli*	5.6 (1.1)	8.2 (4.5)	2.7 (0.7)	3.4 (1.4)
*Clostridia*	3.2 (1.0)	4.1 (1.7)	0.1 (0.1)	0.1 (0.1)
*Cyanobacteria*	14.0 (0.9)	10.0 (2.1)	1.1 (0.1)	1.1 (0.2)
*Planctomycetes*	4.7 (0.6)	4.1 (0.9)	0.8 (0.1)	0.6 (0.2)

Asterisks indicate a significant difference (*P*<0.05; *t*-test) between uninoculated controls (−) and inoculated plants (+). Data for bacterial taxa with low relative abundance (<1%) are not shown. Grey areas highlight the taxa with relative abundance, significantly different between inoculated and uninoculated rice plants.
