# Supplementary material for: Impact of Azospirillum sp. B510 Inoculation on Rice-Associated Bacterial Communities in a Paddy Field
Source: Microbes Environ. 2013 Nov 19;28(4):487–90. doi: 10.1264/jsme2.ME13049 (PMC4070703; doi:10.1264/jsme2.ME13049)
Supplement: Supplementary file 1 [file 28_487_s1.pdf]

Supplementary materials

Impact of *Azospirillum* sp. B510 inoculation on rice-associated bacterial communities in a rice paddy field

Zhihua Bao[1], Kazuhiro Sasaki[1], Takashi Okubo[1], Seishi Ikeda[2], Mizue Anda[1], Eiko Hanzawa[1], Kaori Kakizaki[1], Tadashi Sato[1], Hisayuki Mitsui[1], Kiwamu Minamisawa[1]*

[1]*Graduate School of Life Sciences, Tohoku University, Katahira, Aoba-ku, Sendai, Miyagi 980-8577, Japan;* [2]*National Agricultural Research Center for Hokkaido Region, Shinsei, Memuro-cho, Kasaigun, Hokkaido 082-0081, Japan*

Supplementary Material and Methods

Plate count for internal colonization of the *Azospirillum* sp. strain B510

Surface-sterilized rice seed inoculated (1×10^6 cells/seed) by *Azospirillum* sp. strain B510 carrying the plasmid pHC60 (Cheng and Walker, 1998) that constitutively expresses *gfp* and incubated in test tube containing 20 ml mineral nutrient solution with 0.3 % agar under light (16h)/dark (8h) cycles for 7 days at 25 ℃. After 7 days incubation, the rice tissue were surface sterilized with 70% EtOH and 1% NaOCl for 30 s, respectively. Then, Macerate the tissue with pestle and it was made by serial 10-fold dilution (10^{-1}～10^{-5}) and 0.1 ml of dilutions were transferred to a 0.8xNA agar plate containing Tc (10ug/ml). These plates were incubated at 30℃ for CFU calculation.

Reference

Cheng, H-P and GC. Walker. 1998. Succinoglycan is required for initiation and elongation of infection threads during nodulation of alfalfa by Rhizobium meliloti. J Bacteriol. 180:5183–5191.

Table S1. Numbers of pyrosequece reads (16S rRNA) from base and shoot of rice plants with (+) and without (–) *Azospirillum* sp. B510 inoculation[a]

Sample no.	Number of reads			
	Base		Shoot	
	–	+	–	+
1	11,036	10,084	8276	9236
2	12,027	13,457	4804	4876
3	10,843	3204	4556	2044
Total reads	33,906	26,745	17,636	16,156

Table S2. Relative abundance of bacterial taxa (as determined from 16S rRNA) in rice plant base and shoot with (+) and without (–) *Azospirillum* sp. B510 inoculation[a]

	Relative abundance (%)											
	Base (–)			Base (+)			Shoot (–)			Shoot (+)		
Taxa	1	2	3	1	2	3	1	2	3	1	2	3
Proteobacteria	52.9	49.8	58.8	68.0	54.3	45.6	93.5	90.0	90.4	93.8	89.2	90.1
Alphaproteobacteria	39.9	35.7	39.5	45.5	33.4	31.5	45.3	63.3	58.5	51.0	56.1	58.6
Betaproteobacteria	7.2	8.3	6.6	11.3	13.0	7.8	34.7	18.8	9.5	4.7	16.6	11.6
Gammaproteobacteria	1.8	1.3	9.8	6.8	3.3	3.6	12.5	6.4	21.6	37.1	15.2	16.5
Deltaproteobacteria	1.2	1.4	0.6	1.1	0.9	0.6	0.3	0.5	0.2	0.3	0.2	2.7
others	2.8	3.1	2.3	3.3	3.8	2.1	0.8	1.0	0.6	0.6	1.1	0.6
Firmicutes	10.6	13.1	8.0	9.3	17.8	19.6	2.4	2.3	4.0	1.9	5.5	3.3
Negativicutes	2.3	1.7	1.3	3.9	3.7	2.2		0.1		<0.04		
Bacilli	4.9	7.1	4.8	3.0	7.8	13.9	2.3	2.1	3.7	1.7	5.2	3.3
Clostridia	3.4	4.3	1.9	2.3	6.4	3.5	0.1	0.1	0.2	0.1	0.2	<0.05
Actinobacteria	7.3	5.5	6.3	5.0	6.7	7.0	1.8	4.0	2.5	1.9	3.1	3.3
Cyanobacteria	13.1	15.2	13.6	7.9	9.2	13.0	0.9	1.1	1.3	1.2	0.8	1.3
Planctomycetes	5.1	5.2	3.8	2.9	4.3	5.1	0.6	0.9	0.8	0.4	0.5	0.8
Bacteroidetes	0.1	0.1		0.1	<0.01						<0.03	
Chloroflexi	0.1	<0.03	0.1	0.1	0.1	0.1			0.1		<0.05	0.1
Acidobacteria	0.2	0.2	0.2	0.2	0.4	0.2	<0.05	0.2		0.1	0.3	0.1
Gemmatimonadetes	<0.01		<0.02	<0.01	<0.04			0.1				
Armatimonadetes	0.1		<0.01	<0.01	0.1	<0.04	<0.02					
Verrucomicrobia	1.4	0.8	0.5	1.3	0.8	0.6	<0.03		0.1	0.1		<0.05
BRC1	<0.05			<0.02			<0.02					
TM7	0.2	0.4	0.4	0.1	0.2	0.8	0.1	0.2	0.2	<0.02	<0.03	
Chlorobi	<0.01		<0.02									
Fusobacteria		<0.01						0.5				
other bacteria	8.8	9.7	8.3	5.0	6.1	8.0	0.7	0.7	0.6	0.7	0.5	0.9

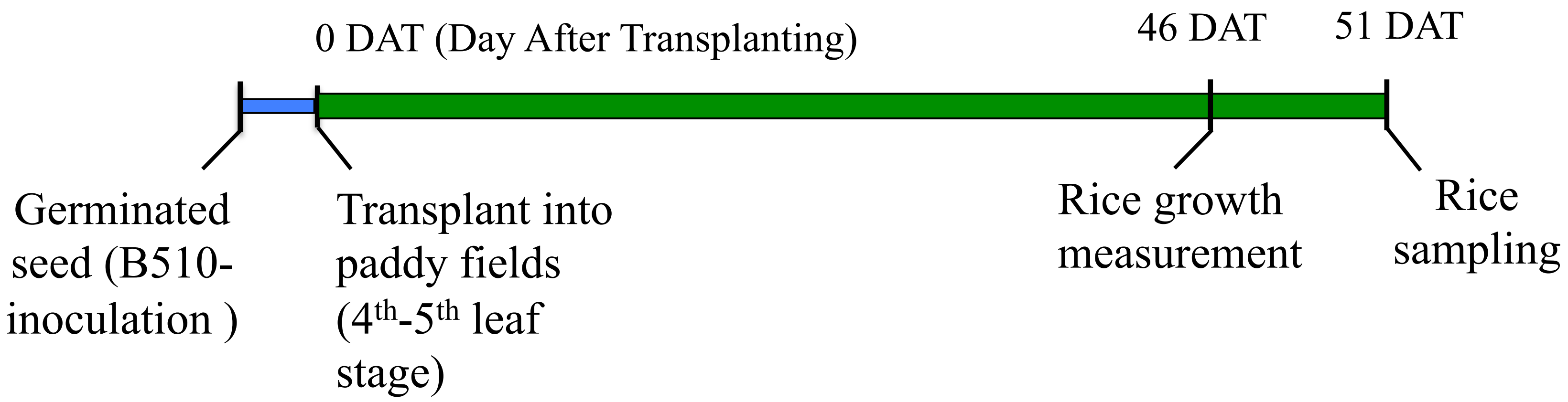

Fig. S1. Schematic showing time course of rice seedling inoculation with *Azospirillum* sp. B510, transplantation, growth measurement, and tissue sampling for bacterial community analysis (tillering stage) in field experiment. Separation of rice plant tissues into base and shoot for sampling are shown in Fig. S2.

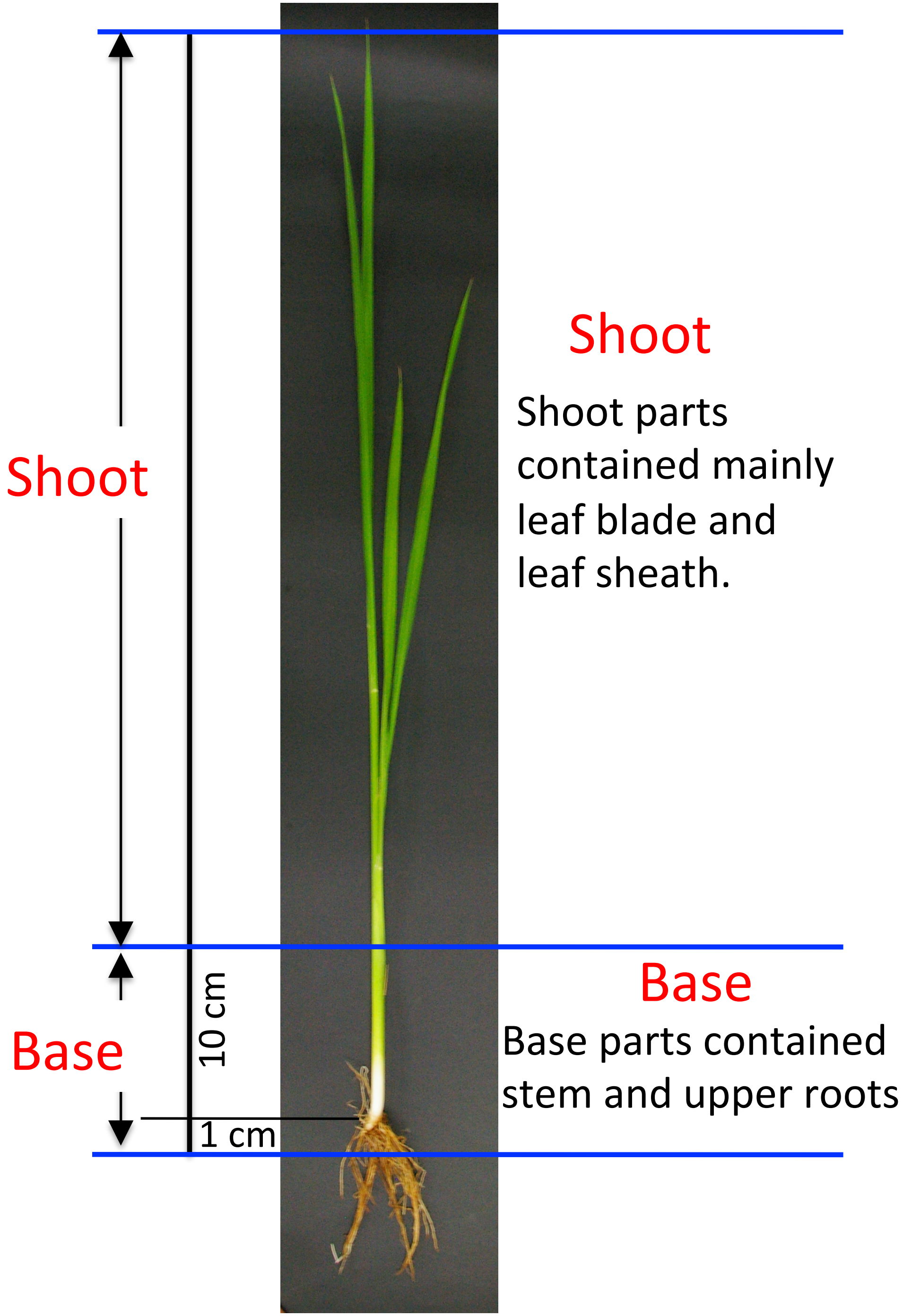

Fig. S2. Rice plants sampled from paddy fields were separated into shoot (main stem and plant above 10 cm from root) and base (bottom 10 cm of stem and top 1 cm of tap root) for plant-associated bacterial community analysis.

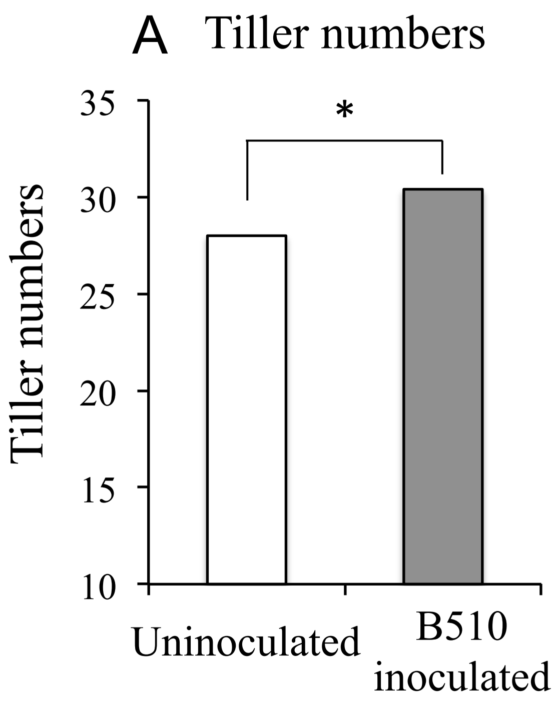

Fig. S3. Effects of inoculation with *Azospirillum* sp. B510 on rice seedling tiller numbers (A) and shoot length (B) after 46 d when planted under standard fertilization. Asterisks indicate significant differences (*t*-test, $P<0.05$; $n=25$). B501 inoculation significantly enhanced tiller numbers (by 8.6%) and shoot length (3.1%) as compared with uninoculated controls.
